# Effects of Microbial Communities on Volatile Profiles and Biogenic Amines in Beef Jerky from Inner Mongolian Districts

**DOI:** 10.3390/foods11172659

**Published:** 2022-09-01

**Authors:** Xueying Sun, Lina Sun, Lin Su, Huiting Wang, Dan Wang, Jianlin Liu, Erke Sun, Guanhua Hu, Chang Liu, Aiwu Gao, Ye Jin, Lihua Zhao

**Affiliations:** College of Food Science and Engineering, Inner Mongolia Agricultural University, Hohhot 010018, China

**Keywords:** beef jerky, microbial community, biogenic amine, volatile compounds

## Abstract

Beef jerky is a traditional fermented meat product from Inner Mongolia, handcrafted by artisans. We investigated the bacteria of the microbial community, volatile flavor components, and biogenic amines of Inner Mongolia beef jerky via high-throughput sequencing, solid-phase microextraction with gas chromatography–mass spectrometry, and high-performance liquid chromatography, respectively. Thirty-three bacteria were identified, predominantly from the genera *Pseudomonas* (45.4%), *Ralstonia* (13.4%), and *Acinetobacter* (7.3%). Fifty-nine volatile flavor compounds and eight biogenic amines were detected. Based on Spearman’s correlation coefficient, 20 bacterial genera were significantly associated with the dominant volatile compounds in the beef jerky samples (*p* < 0.05). The results demonstrated that beef jerky may be toxic due to cadaverine, putrescine, and histamine; moreover, the amounts of putrescine and cadaverine were positively correlated with the abundance of *unclassified_f_Enterobacteriaceae* (*p* < 0.05). These findings shed light on the formation of the microbial community, flavor components, and biogenic amines of beef jerky, thereby providing a basis for improving its quality.

## 1. Introduction

Beef jerky, a traditional dried meat product, is favored by people in China because of its slow deterioration (and consequent suitability for long-term storage) and unique flavor [1]. The production process of beef jerky is generally to remove fascia and fat from beef, and the meat is divided into large pieces along the direction of meat fiber, which is usually produced by spontaneous fermentation and drying. The finished product is then packaged. The bacteria that grow in beef jerky are mainly found in raw beef and the production environment; they constitute a unique community that increases the quality of the product and helps preserve it [2,3]. In China, beef jerky is mainly processed in the provinces of Inner Mongolia, Liaoning, Sichuan, and Henan. Beef jerky is the most popular and notable product from Inner Mongolia [4]. The fermentation of jerky occurs in an open environment containing many types of microorganisms. The microorganisms in jerky may differ depending on the country of origin, recipe, and temperature. Meat products are mainly inoculated with *Lactobacillus*, *Enterobacteria*, *Leuconostoc*, and *Pseudomonas* [5,6,7]. 

The composition of the microbial community has a major influence on the characteristics and taste of jerky, which develops its unique flavor during the spontaneous fermentation of meat. There are several influential factors in this process, such as the type of raw materials, the amount of additives, the quantity of proteins, and lipids [8]. Hydrolysis of proteins and lipids causes the release of amino acids and fatty acids, which are used by microorganisms as food [9]. Microbiological enzymes degrade free fatty acids into short-chain fatty acids through lipid β-oxidation reactions; these short-chain fatty acids contribute to the pungent smell of meat [10]. During Strecker degradation, sulfur-containing amino acids produce sulfur compounds that form hydrogen sulfide through free radical reactions, giving them a pungent taste and odor [11]; free amino acids are decarboxylated and deaminated by microorganisms, forming ammonia [12].

A biogenic amine (BA) is an alkaline compound containing nitrogen that has a low molecular weight [13]. Microbial enzymes decarboxylate free amino acids in meat products [14,15]. Cells depend on BAs for their vital functions, and the body continually synthesizes and catabolizes these amines to maintain normal concentrations in cells and tissues [16]. Excess exogenous intake can upset the balance of BAs and cause various diseases, including food poisoning and cancer [17]. Excessive free amino acids affect decarboxylase activity and bacterial growth, with many studies reporting that fermented meat products contain high concentrations of BAs [18]. Papavergou and Ekaterini detected approximately 0–492 and 4–381 mg/kg of putrescine (PUT) and tyramine, respectively, in sausages, and 37% of the sausage samples were considered to pose a risk of histamine poisoning (>50 mg/kg) [19]. Studying metabolites and microbial composition is vital to understanding their roles [20].

Only a few reports have identified BA content and volatile flavor compounds in jerky sold on the Chinese market. Thus, the purpose of this study is to examine the types and levels of BAs and volatile flavor compounds present in beef jerky produced in different regions of Inner Mongolia and to assess microbial community diversity. In summary, we investigated the relationships among BAs, volatile flavor compounds, and microbiota in beef jerky.

## 2. Materials and Methods

### 2.1. Sample Collection

Sixteen samples of beef jerky were made and collected from local producers in eight cities in Inner Mongolia (Xilin Hot, Hulun Buir, Xing’an league, Alxa league, Tongliao, Ulanqab, Chifeng, and Hohhot) in 2020. All collected samples were vacuum-packed and stored at −80 °C before being transported.

### 2.2. High-Throughput Sequencing

#### 2.2.1. DNA Extraction and PCR Amplification

We extracted genomic DNA from the beef jerky samples according to the methods of Wang et al. [21]. A 1% agarose gel and ultraviolet–visible spectrophotometer (NanoDrop 2000; Thermo Scientific, Wilmington, DE, USA) were used to quantify the DNA concentration and purity. Taking hypervariable region V3–V4 as an example, we used primer pairs 338F (5′-ACTCCTACGGGAGGGATG-3′) and 806R (5′-GGACTACHVGGGTWTCTAAT-3′) of the bacterial 16S rRNA gene and a PCR thermocycler (GeneAmp 9700; Applied Biosystems, Foster City, CA, USA). To amplify the 16S rRNA gene, it was denatured at 95 °C for 3 min, and then at 95 °C for 30 s for 27 cycles. Then, it was annealed at 58 °C for 30 s, with an extension at 72 °C for 30 s, and a final extension at 72 °C for 5 min. The reaction was terminated at 4 °C. The PCR mixtures included the following components: 5 × 4 μL of FastPfu buffer (TransStart; TransGen Biotech, Beijing, China), 2 μL of 2.5 mM deoxyribonucleoside triphosphates, 0.8 μL (5 μM) each of forward and reverse primers, 0.4 μL of FastPfu DNA Polymerase (TransStart), and 10 ng of template DNA (adjusted to 20 μL with double-distilled water). Each PCR reaction was performed in triplicate. We purified the PCR product from 2% agarose gel using a DNA gel extraction kit (AxyPrep; Axygen Biosciences, Union City, CA, USA). A fluorometer (Quantus; Promega, Madison, WI, USA) was used to quantify the samples following the manufacturer’s instructions.

#### 2.2.2. Sequencing and Data Analysis

The amplicons were sequenced on the MiSeq PE300/NovaSeq PE250 platforms (Illumina, San Diego, CA, USA) by a commercial laboratory (Majorbio Bio-Pharm Technology Co., Ltd., Shanghai, China) according to standard protocols. The data were de-multiplexed and quality-filtered using Fastp software (version 0.20.0, Shenzhen, China).

### 2.3. Determination of Volatile Flavor Compounds

The volatile flavor compounds in beef jerky were measured by gas chromatography–mass spectrometry (GC–MS); 5 g of jerky was minced and placed in 20 mL headspace vials and then equilibrated and extracted for 40 min at 60 °C. Volatile compounds were measured in a capillary column (TR-5; 30 m × 0.25 mm × 0.25 μm) using a gas chromatograph (Trace 1300, ISQ series; Thermo Fisher, Waltham, MA, USA) linked to a solid phase microextraction detector. Samples were heated at 35 °C for 5 min, then to 130 °C at a rate of 4 °C/min, and 200 °C at a rate of 10 °C/min.

Thermal desorption of volatile flavor compounds was performed with the GC injection port at a temperature of 250 °C. The temperature of the GC column was initially set to 40 °C for 3 min, increased to 150 °C at a rate of 4 °C/min, held for 1 min, increased to 200 °C at a rate of 5 °C/min, and finally increased to 230 °C at a rate of 20 °C/min and held for 5 min. Helium (99.999%) was used as the carrier gas, flowing at a constant flow rate of 1 mL/min; the ionization voltage was 70 eV. with a mass range of 30–400 (*m*/*z*) for the GC–MS chromatograms. Electron ionization was used as the ionization method. To prevent solvent peak interference, a solvent delay of 1.0 min was set; peaks in the GC–MS chromatograms were identified by searching the MEANLIB, Nist Demo, and Willey libraries. We accepted any match with a score of ≥800 [22]. All compounds were semi-quantified as 2-methyl-3-heptanone equivalents [23]. The volatile compounds were analyzed in triplicate (*n* = 3). The compounds examined were alcohols, esters, acids, aldehydes, volatile phenols, and ketones.

### 2.4. Biogenic Amine Analysis

To obtain BAs from beef jerky, we followed the protocol of Lu et al. with some minor modifications [24]. Samples (approximately 5 g) were blended with 20 mL of 0.4 M perchloric acid and extracted repeatedly, with the filter liquor volume made up to 50 mL with 0.4 M perchloric acid, and then centrifuged (5810R; Eppendorf, Hamburg, Germany) at 5000× *g* for 10 min at 4 °C. Then, 1 mL of the sample extract was added to 200 µL of 2 M sodium hydroxide, 300 µL of saturated sodium bicarbonate, 2 mL of 10 mg/mL dansyl chloride, and reacted at 40 °C in the dark.

After standing for 45 min at ambient temperature, 100 µL of ammonia was used to remove the residual dansyl chloride, followed by a 30-min incubation at room temperature. The reaction mixture was made up to 5 mL with acetonitrile and centrifuged at 3000× *g* for 5 min. Then, 20 µL of the filtrate from each beef jerky sample was filtered for analysis by high-performance liquid chromatography (1260; Agilent, Santa Clara, CA, USA). As part of this analysis, a chromatographic column (ZORBAX SB-C18; Agilent) was filled with the sample at a flow rate of 0.9 mL/min using an ultraviolet detection wavelength of 254 nm. Acetonitrile was the first solvent used in the elution process, followed by water (solvent B). The parameters during the 0–5 min period were as follows: mobile phase A, 35–25%; and mobile phase B, 65–75%. During the 44–55 min period, they were as follows: mobile phase B, 100%; mobile phase A, 35%; and mobile phase B, 65%.

### 2.5. Statistical Analysis

Spearman correlation was used to assess the relationship between the microbiota and BA concentrations. Flavor components were visualized using IBM SPSS software (version 22.0; IBM Corp., Armonk, NY, USA) and figures were drawn with TBtools software (version 1.098722, Guangdong, China). Principal component analysis and partial least squares discriminant analysis were performed using SIMCA software (version 13.0; Umetrics, Umea, Sweden). A total of *p* < 0.05 was taken to indicate statistical significance in the experiments, all of which were performed in triplicate. Data are expressed as mean ± standard deviation.

## 3. Results and Discussion

### 3.1. Bacterial Community Composition of Beef Jerky from Different Areas of Inner Mongolia

In total, we obtained 2,697,627 high-quality sequence reads of V3–V4 16S rRNA genes from the 16 samples (range: 38,659–75,682), having a length of 425 bp. The results revealed that 659 ± 294 bacterial operational taxonomic units (OTUs) with a similarity level of 97%. The coverage values were all ≥99%, indicating that the sequencing reads adequately represented the microbial diversity of the samples [25].

Table 1 lists the values of many alpha-diversity indices for the 16 samples, including observed reads, OTUs, abundance-based coverage estimators (ACE), and the Chao1, Simpson, Shannon, and Goods coverage indices. The Shannon and Simpson indexes can reveal the microbial diversity of beef jerky, with microbial diversity generally being higher in environments with a high Shannon index; the opposite is true for the Simpson index [26]. The Chao1 index is similar to the ACE index, which is used to estimate the total number of species in samples. In this study, the ACE and Chao1 indexes of samples S1 and S2 from Chifeng were higher than those of the other samples, indicating greater species diversity. All of the beef jerky samples, except for S10 and S14, had high Chao1 values (>300). S13 and S14 had the lowest Shannon indexes (0.69 and 0.62, respectively).

**Figure 1:**
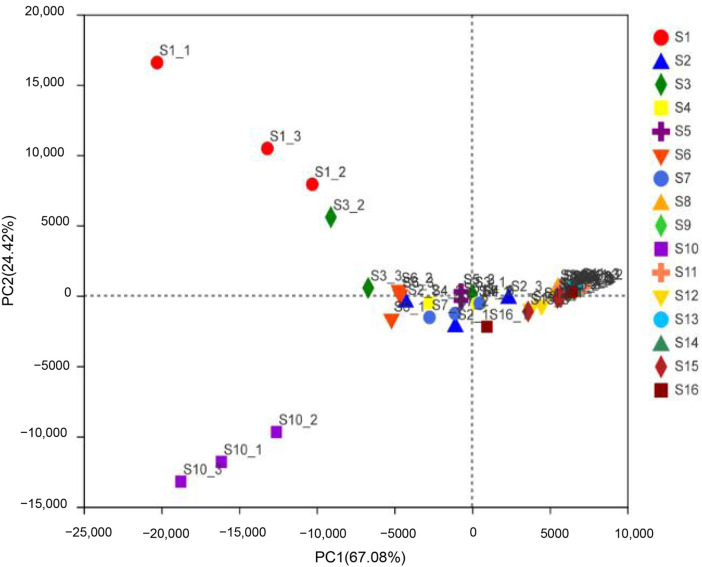
Cluster analysis of bacterial community composition based on principal coordinate analyses (PCoAs) of the beef jerky samples.

In the present study, 16 samples of beef jerky were compared across regions in Inner Mongolia in terms of their alpha diversity. A clustering pattern of microbial communities was identified. We conducted a principal coordinates analysis based on the relative abundance of the different OTUs. The results revealed that the samples from different areas of Inner Mongolia could be divided into three main clusters (Figure 1), suggesting that the microbial community composition was probably affected by location. The study shows that the bacterial composition of the product depends on the production process and conditions as well as the bacteria in the air. The production conditions of the products are different according to the regional preference and temperature. Therefore, the bacterial composition and function of the product may be substantially different in the different provinces in Korea [27].

**Figure 2:**
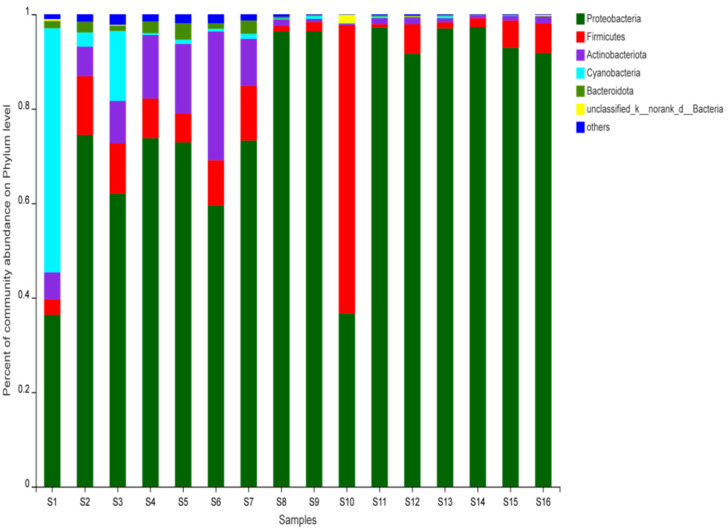
Relative abundance of bacteria at the phylum level in the samples.

**Figure 3:**
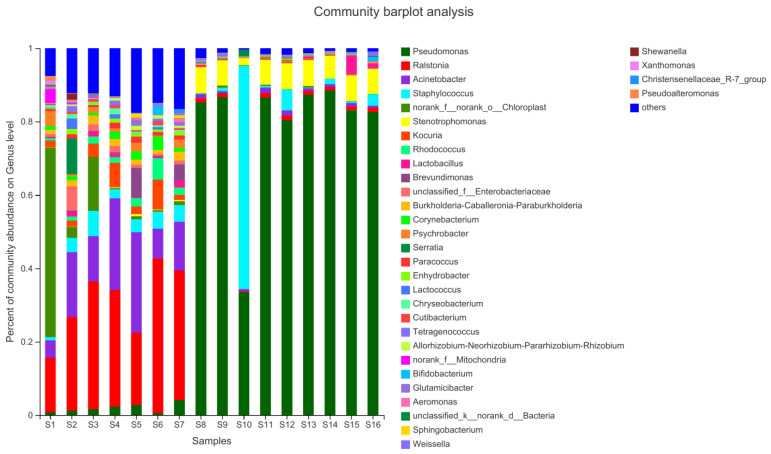
Relative abundance of bacteria at the genus level in the samples.

The results showed that Proteobacteria were the most common bacteria (78%). S1, S6, and S10 contained higher amounts of Cyanobacteria, Actinobactenota, and Firmicutes, respectively (Figure 2). Thirty-three genera were identified at the genus level (average relative abundance >1%), and their relative abundances in the jerky are shown in Figure 3. The most common bacterial genera in the 16 samples were Kocuria (1.7%), Staphylococcus (6.3%), Pseudomonas (45.4%), Ralstonia (13.4%), Acinetobacter (7.3%), and Stenotrophomonas (3.5%). Pseudomonas was more common in S8, S9, S11, S12, S13, S14, S15, and S16.

### 3.2. Composition of Volatile Flavor Compounds of Beef Jerky from Different Areas of Inner Mongolia

**Figure 4:**
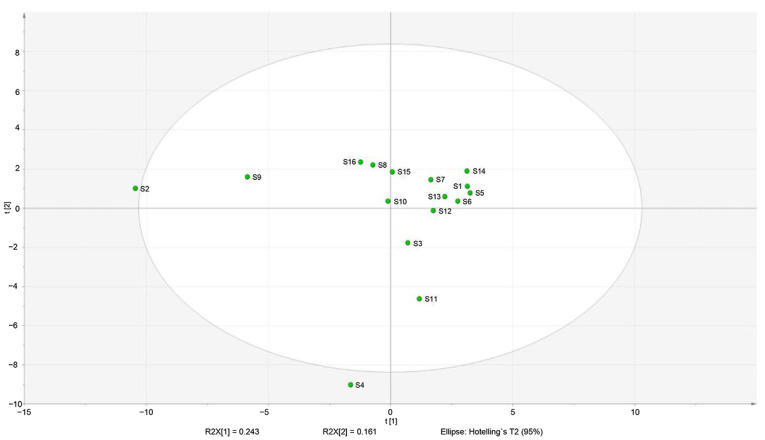
PCA results at the beef jerky samples of 59 volatiles.

**Figure 5:**
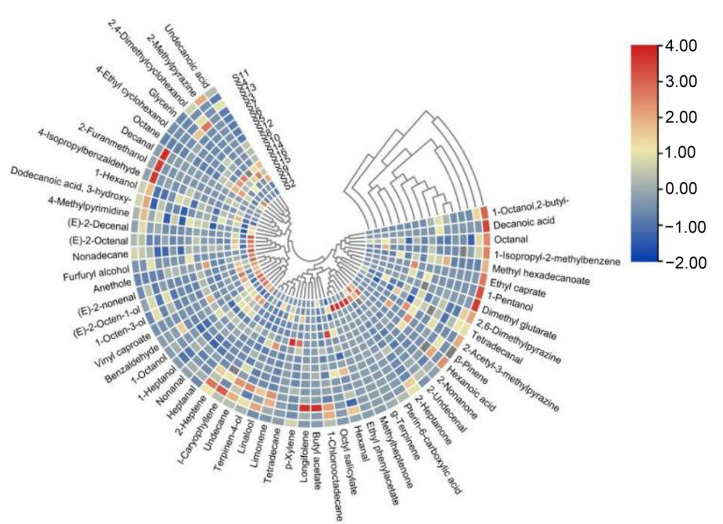
Heat map cluster analysis of the volatile components with the Euclidean distance and McQuitty’s criterion.

The taste and quality of beef jerky are primarily determined by its flavor profile. The results of the principal component analysis of the volatile components in our samples are shown in Figure 4. Principal components 1 and 2 differed significantly among the sample groups. Table S1 lists the concentrations of volatile compounds found in the beef jerky samples. In total, 59 volatile compounds, including 14 alcohols, 12 aldehydes, 7 esters, 6 olefins, 5 acids, 5 alkanes, 3 ketones, and 7 others were identified and quantified. The 16 samples were classifiable into 4 volatile compound categories (Figure 5). Twelve of the compounds, including decanal, 2-furanmethanol, and 4-isopropylbenzaldehyde, were in the first category and were prominent in S4. There were 14 compounds in the second category with substantial flavor diversity; (E)-2-octen-1-ol, 1-octen-3-ol, 1-octanol, (E)-2-nonenal, (E)-2-decenal, alcohol, and aldehydes were the most abundant. The compounds in this category were most abundant in S2 and S9. There were 20 compounds in the third category, including 2 alcohols, 3 esters, 2 aldehydes, 3 ketones, 5 olefins, and 5 others. Esters were predominant in the fourth category, including dimethyl glutarate, ethyl caprate, and methyl hexadecanoate in S11. These results suggest that metabolites differ among beef jerky produced in different regions of Inner Mongolia. Some researchers found similar results [4].

### 3.3. Biogenic Amine Concentrations of Beef Jerky from Different Areas of Inner Mongolia

Table 2 lists the concentrations of BAs measured in the samples. Tyramine was only found in 8 out of 16 samples, in amounts ranging from 17.85–86.19 mg/kg (i.e., below the European Union’s maximum level of 100 mg/kg). Concentration ranges of 31.66–62.48, 0–63.31, and 0–72.2 mg/kg were observed for tryptamine, 2-phenylethylamine, and spermidine, respectively. In the samples from Chifeng, the only metabolites detected at >100 mg/kg were PUT (279.56 mg/kg), cadaverine, histamine, and spermine, although these were also observed in some samples at levels <100 mg/kg. Among the 16 samples of beef jerky examined for BAs, S2 had the highest concentration (1691.73 mg/kg).

### 3.4. Correlation of Volatile Compound Composition with Microbial Community

**Figure 6:**
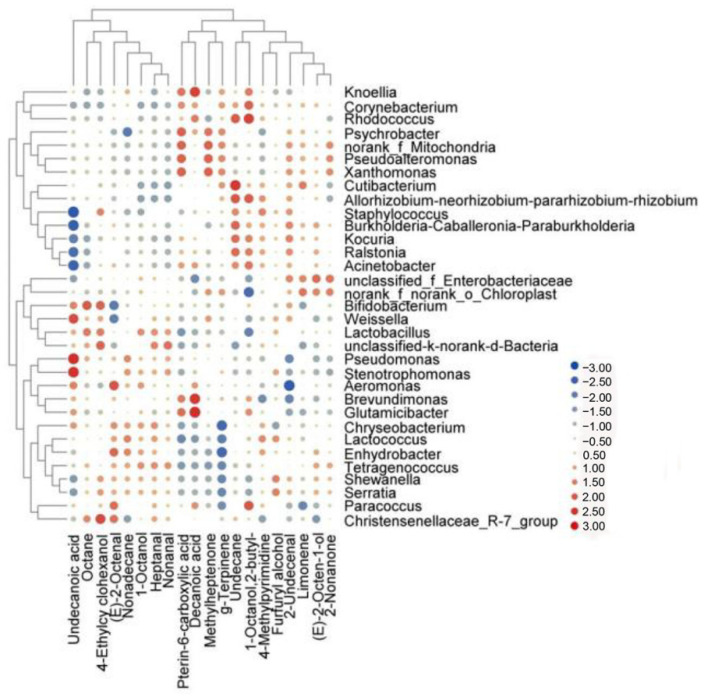
Heat map of the correlation between flavor compounds and microorganisms of beef jerky.

A meat product’s flavor is determined by microorganisms, which grow and (The detection limits of 8 kinds of biogenic amines were less than 0.5 mg/kg) metabolize [28]. We quantified the relationships among 33 microbes and 20 volatile flavor compounds using Spearman’s correlation coefficient. As shown in Figure 6, there was a strong correlation between 20 bacterial genera and the volatile compounds in the beef jerky samples (*p* < 0.05). The accumulation of methylheptenone and pterin-6-carboxylic acid was positively associated with changes in *Pseudoalteromonas*, *Xanthomonas*, and *norank_f_Mitochondria* (*p* < 0.05). *Enhydrobacter* had significant positive correlations with (E)-2-octenal and g-Terpinene (*p* < 0.05). *Tetragenococcus* was positively correlated with heptanal, (E)-2-octenal, (E)-2-octen-1-ol, 2-nonanone, nonadecane, 1-octanol, and nonanal (*p* < 0.05). *Unclassified_f_Enterobacteriaceae* had significant positive correlations with limonene, (E)-2-octen-1-ol, 2-nonanone, and 2-undecenal (*p* < 0.05). *Lactobacillus* was associated with octane, 4-ethylcy clohexanol, 1-octanol, and 2-butyl. Generally, Lactobacillus is associated with high levels of volatile components, such as aldehydes, alcohols, and esters [29,30].

**Figure 7:**
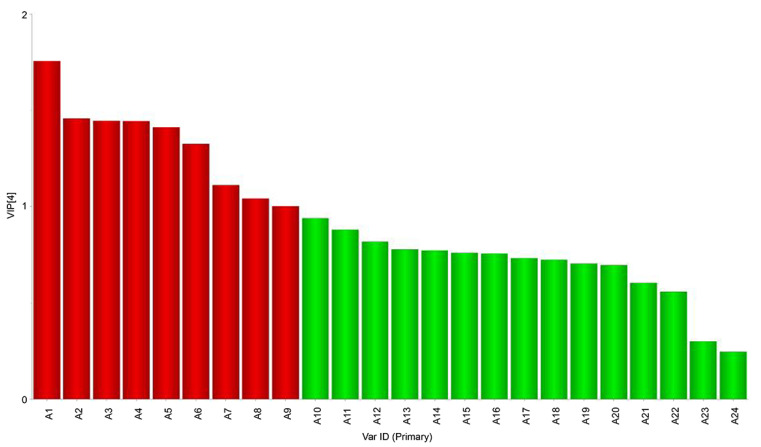
Analysis of the microbiota and volatile compounds using a VIP plot of bacteria and volatile substances (A1: *norank_f_norank_o_Chloroplast*; A2: *Psychrobacter*; A3: *Tetragenococcus*; A4: *Lactococcus*; A5: *unclassified_f_Enterobacteriaceae*; A6: *Enhydrobacter*; A7: *Chryseobacterium*; A8: *Rhodococcus*; A9: *Brevundimonas*; A10: *Kocuria*; A11: *Corynebacterium*; A12: *Paracoccus*; A13: *Acinetobacter*; A14: *Pseudomonas*; A15: *Ralstonia*; A16: *Stenotrophomonas*; A17: *Cutibacterium*; A18: *Glutamicibacter*; A19: *Burkholderia-Caballeronia-Paraburkholderia*; A20: *Lactobacillus*; A21: *Aeromonas*; A22: *Bifidobacterium*; A23: *unclassified-k-norank-d-Bacteria*; A24: *Staphylococcus*).

We identified microbial communities closely related to volatile flavor compounds [active (x) and attribute (y) variables, respectively]. Microorganisms influencing the formation of volatile flavor compounds also affect their variable importance (VIP) values, which denote their power to explain the results [31,32]. The VIP values in this study are shown in Figure 7; bacteria with values ≥ 1 are marked in red; all others are in green. Based on this criterion, we identified nine bacterial genera, i.e., *norank_f_norank_o_Chloroplast*, *Psychrobacter*, *Tetragenococcus*, *Lactococcus*, *unclassified_f_Enterobacteriaceae*, *Enhydrobacter*, *Chryseobacterium*, *Rhodococcus*, and *Brevundimonas*, which likely have a significant impact on taste.

### 3.5. Correlation of Biogenic Amine Concentration with Microbial Community

**Figure 8:**
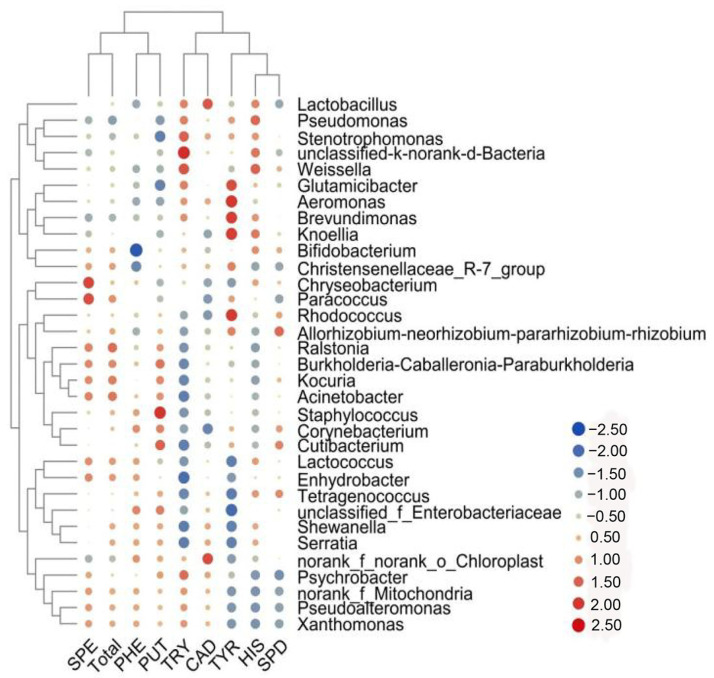
Heat map of the correlation between biogenic amines and microorganisms of beef jerky.

We revealed the role of microbes in the production of BAs in beef jerky by analyzing the correlations between bacterial communities and BA concentrations. Significant results were obtained for 11 bacteria and 7 BAs. As shown in Figure 8, PUT showed a strong positive correlation with *unclassified_f_Enterobacteriaceae* and *Staphylococcus*. 2-phenylethylamine and cadaverine showed positive relationships with *unclassified_f_Enterobacteriaceae* and *norank_f_norank_o_Chloroplast*, respectively. The results of our study show that bacteria can produce BAs [33].

**Figure 9:**
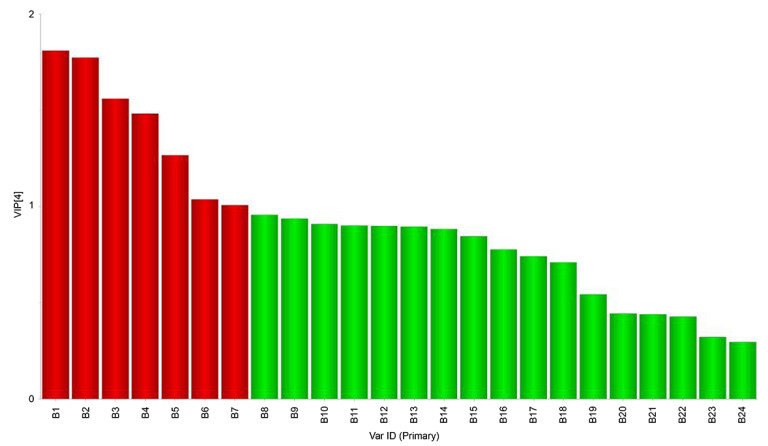
Analysis of the microbiota and biogenic amines using a VIP plot of bacteria and volatile substances (B1: *unclassified_f_Enterobacteriaceae*; B2: *Lactococcus*; B3: *Enhydrobacter*; B4: *Tetragenococcus*; B5: *Chryseobacterium*; B6: *Kocuria*; B7: *Rhodococcus*; B8: *Pseudomonas*; B9: *Stenotrophomonas*; B10: *Acinetobacter*; B11: *Ralstonia*; B12: *Corynebacterium*; B13: *Psychrobacter*; B14: *Bifidobacterium*; B15: *Burkholderia-Caballeronia-Paraburkholderia*; B16: *Cutibacterium*; B17: *norank_f_norank_o_Chloroplast*; B18: *Paracoccus*; B19: *Brevundimonas*; B20: *Aeromonas*; B21: *Glutamicibacter*; B22: *Lactobacillus*; B23: *unclassified-k-norank-d-Bacteria*; B24: *Staphylococcus*).

By calculating and measuring the VIP values, we were able to determine the explanatory power of each microorganism with respect to the formation of BAs. Seven genera had a VIP value > 1, including *Lactococcus*, *Lactococcus*, and *Tetragenococcus* (Figure 9), which suggests that they have significant effects on amine production.

## 4. Conclusions

We performed this study to determine the relationships among microorganisms, BAs, and volatile flavor compounds found in beef jerky. Analyses of the diversity of bacteria, flavor quality, and BAs were conducted. We also aimed to characterize the structure of the microbial community. A total of 33 bacteria genera were detected. In total, 59 volatile compounds, including 14 alcohols, 12 aldehydes, 7 esters, 6 olefins, 5 acids, 5 alkanes, 3 ketones, and 7 others, were identified and quantified. Spearman’s correlation revealed that flavor compounds were related to *Psychrobacter*, *Tetragenococcus*, *Lactococcus*, and *Enhydrobacter*. High-performance liquid chromatography revealed that S2 contained the highest level of PUT (269.66 mg/kg); PUT positively correlates with *unclassified_f_Enterobacteriaceae* and *Staphylococcus*. These findings have increased our understanding of the core microbiota related to the volatile profiles and biogenic amines of beef jerky. The present study could aid the production of safer beef jerky and provide a theoretical basis for improving its flavor. However, further studies are needed to evaluate microbial metabolism and activity using metatranscriptomics.

## Figures and Tables

**Table 1:** Statistical analysis of the sequencing data sets from the beef jerky samples.

Sample	Total Reads	OTUs	Shannon	Simpson	ACE	Chao1	Goods Coverage
S1	55,861	1068	2.39	0.32	696.01	639.2	99.52
S2	49,546	1133	3.43	0.10	634.08	639.09	99.62
S3	53,181	1051	3.10	0.16	538.74	541.76	99.75
S4	51,515	1039	3.16	0.14	601.34	608.36	99.64
S5	55,592	730	3.45	0.12	413.35	414.46	99.83
S6	75,682	1083	3.02	0.20	561.98	563.54	99.70
S7	46,200	928	3.45	0.14	536.29	539.23	99.75
S8	65,063	790	0.85	0.73	579.19	478.76	99.55
S9	60,658	518	0.74	0.75	546.15	462.00	99.60
S10	73,426	281	1.06	0.47	290.19	257.00	99.83
S11	46,579	399	0.73	0.75	373.79	311.64	99.71
S12	57,201	584	1.00	0.65	437.79	356.04	99.66
S13	57,823	477	0.69	0.76	388.62	335.63	99.66
S14	38,659	230	0.62	0.78	251.29	201.42	99.81
S15	57,355	416	0.83	0.70	477.39	340.92	99.68
S16	56,819	467	0.91	0.67	490.42	341.02	99.68

Abbreviations: OTUs, operational taxonomic units.

**Table 2:** Biogenic amine concentration in jerky (mg/kg).

mg/kg	TRY	PHE	PUT	CAD	HIS	TYR	SPD	SPE	Total
S1	62.48 ± 3.93 ^a^	33.55 ± 10.16 ^b^	68.99 ± 8.23 ^b^	87.64 ± 8.6 ^c^	47 ± 1.88 ^bcd^	-	-	351.75 ± 97.75 ^a^	651.41 ± 96.89 ^b^
S2	40.65 ± 0.53 ^fg^	63.31 ± 12.29 ^a^	269.66 ± 65.73 ^a^	867.61 ± 163.53 ^a^	157.65 ± 27.05 ^a^	-	34.11 ± 0.54	258.75 ± 81.46 ^bc^	1691.73 ± 350.04 ^a^
S3	41.24 ± 3.26 ^fg^	16.21 ± 0.67 ^de^	59.19 ± 9.64 ^b^	63.86 ± 6.05 ^c^	43.46 ± 2.78 ^d^	-	-	132.27 ± 43.66 ^f^	350.82 ± 61.21 ^def^
S4	31.66 ± 0.31 ^h^	24.36 ± 2.78 ^bcd^	65.25 ± 18.02 ^b^	59.32 ± 12.34 ^c^	61.14 ± 24.72 ^bcd^	-	52.62 ± 8.5 ^ab^	268.73 ± 61.36 ^bc^	563.08 ± 78.79 ^bc^
S5	42.07 ± 2.4 ^fg^	20.32 ± 3.43 ^cde^	56.26 ± 3.15 ^b^	59.07 ± 13.19 ^c^	55.93 ± 8.6 ^bcd^	17.85 ± 2.24 ^e^	-	172.14 ± 23.88 ^def^	423.65 ± 25.01 ^cdef^
S6	31.85 ± 0.47 ^h^	-	59.5 ± 0.46 ^b^	59.42 ± 0.57 ^c^	46.58 ± 1.24 ^cd^	58.19 ± 5.83 ^b^	72.2 ± 33.4 ^a^	243.04 ± 37.37 ^cd^	527.32 ± 58.06 ^bcd^
S7	50.71 ± 4.34 ^cde^	-	57.78 ± 1.45 ^b^	77.93 ± 3.96 ^c^	51.44 ± 3.33 ^bcd^	38.96 ± 0.39 ^cd^	-	252.3 ± 48.84 ^bcd^	516.14 ± 72.26 ^bcde^
S8	56.03 ± 9.73 ^abc^	-	-	179.48 ± 83.53 ^b^	-	44.56 ± 13.5 ^bc^	-	327.6 ± 43.64 ^ab^	498.46 ± 180.96 ^bcde^
S9	47.08 ± 3.84 ^def^	23.26 ± 2.35 ^cd^	57.5 ± 0.59 ^b^	80.71 ± 1.83 ^c^	58.04 ± 2.12 ^bcd^	18.09 ± 1.48 ^e^	35.4 ± 0.74 ^bc^	-	308.29 ± 11.9 ^f^
S10	57.18 ± 6.32 ^abc^	28.1 ± 1.87 ^bc^	70.76 ± 10.46 ^b^	79.7 ± 1.86 ^c^	63.49 ± 1.21 ^bcd^	28.83 ± 12.91 ^cde^	34.15 ± 14.29 ^bc^	173.55 ± 9.04 ^def^	477.92 ± 78.59 ^bcdef^
S11	46.02 ± 0.05 ^def^	11.22 ± 0.35 ^e^	47.16 ± 0.13 ^b^	52.33 ± 0.38 ^c^	71.04 ± 0.45 ^bc^	86.19 ± 6.04 ^a^	0 ± 0	128.34 ± 0.1 ^f^	442.29 ± 5.28 ^cdef^
S12	50.43 ± 6.1 ^cde^	23.73 ± 1.44 ^cd^	55.14 ± 4.46 ^b^	69.9 ± 6.81 ^c^	59.49 ± 5.13 ^bcd^	27.74 ± 4.98 ^de^	0 ± 0	223.24 ± 9.23 ^cde^	509.67 ± 12.8 ^bced^
S13	43.11 ± 2.19 ^efg^	-	50.07 ± 2.95 ^b^	63.68 ± 3.12 ^c^	52.74 ± 3.26 ^bcd^	-	12.43 ± 1.41 ^c^	115.19 ± 6.81 ^f^	333.08 ± 11.99 ^ef^
S14	37.55 ± 0.6 ^gh^	28 ± 3.33 ^bc^	57.25 ± 11.61 ^b^	59.13 ± 3.79 ^c^	70.63 ± 27.66 ^bc^	-	-	153.03 ± 25.04 ^ef^	405.58 ± 35.08 ^cdef^
S15	52.9 ± 1.11 ^bcd^	-	56.19 ± 1.96 ^b^	74.14 ± 1 ^c^	71.44 ± 3.21 ^bc^	-	-	220.5 ± 9.89 ^cde^	475.17 ± 11.54 ^bcdef^
S16	60.48 ± 1.39 ^ab^	-	52.6 ± 1.2 ^b^	62.38 ± 2.66 ^c^	71.97 ± 9 ^b^	-	-	219.07 ± 13.66 ^cde^	466.5 ± 7.02 ^bcdef^

”-” means not detected, ^a^^–h^: Mean values followed different lowercase letters in the same column indicate a significant difference (*p* < 0.05).

## Data Availability

The data presented in this study are available on request from the corresponding author.

## Supplementary Materials

The following supporting information can be downloaded at: https://www.mdpi.com/article/10.3390/foods11172659/s1, Table S1: Concentrations of volatile compound during jerky (μg/kg).
