# Supplementary material for: Effects of Microbial Communities on Volatile Profiles and Biogenic Amines in Beef Jerky from Inner Mongolian Districts
Source: Foods. 2022 Sep 1;11(17):2659. doi: 10.3390/foods11172659 (PMC9455903; doi:10.3390/foods11172659)
Supplement: Supplementary file 1 [file foods-11-02659-s001.zip › foods-1876243-supplementary.pdf]

foods

MDPI

Table S1. Concentrations of volatile compound during jerky (μg/kg).

Volatile compound	S1	S2	S3	S4	S5	S6	S7	S8	S9	S10	S11	S12	S13	S14	S15	S16
**Alcohols**																
1-Pentanol	-	-	-	-	-	-	-	-	-	0.18±0.03	0.5±0.15	0.21±0.01	-	-	-	-
2-Furanmethanol	-	-	-	5.85±1.11	-	-	0.29±0.04	-	-	-	0.51±0.04	-	-	-	1.4±0.24	1.33±0.26
Furfuryl alcohol	-	1.45±0.25	-	-	-	-	-	-	-	-	-	0.6±0.05	0.54±0.02	-	-	-
1-Hexanol	-	-	-	0.71±0.11	0.42±0.09	0.49±0.03	0.15±0.05	0.35±0.07	0.71±0.1	0.26±0.06	0.43±0.12	-	-	-	0.29±0.07	0.35±0.03
1-Heptanol	-	2.24±0.3	0.65±0.03	-	0.24±0.04	-	0.57±0.05	0.31±0.03	1.11±0.18	0.81±0.17	0.39±0.01	0.46±0.03	-	-	0.87±0.19	1.16±0.27
1-Octen-3-ol	-	10.19±1.49	3.81±0.68	6.8±1.06	1.18±0.03	-	-	4.26±0.74	13.81±2.43	0.79±0.17	-	-	-	0.43±0.09	0.8±0.14	1.83±0.39
4-ethyl cyclohexanol	-	0.89±0.33	-	-	-	-	0.15±0.01	-	-	0.25±0.05	-	-	-	-	0.23±0.05	0.79±0.11
Glycerin	-	-	0.59±0.04	-	-	-	-	0.32±0.06	0.45±0.06	0.16±0.02	-	-	-	-	0.28±0.06	0.83±0.2
trans-2-Octen-1-ol	1.02±0.21	3.04±0.18	1.72±0.35	1.62±0.2	0.16±0.03	0.34±0.01	-	1.1±0.17	2.53±0.29	0.33±0.05	0.71±0.14	0.19±0.01	-	-	0.27±0.06	0.65±0.07
1-Octanol	1.33±0.19	5.66±0.75	1.64±0.28	2.2±0.02	0.67±0.12	0.57±0.01	1.58±0.34	3.23±0.57	3.18±0.54	2.02±0.1	1.65±0.35	1.42±0.22	1.54±0.31	0.59±0.02	2.81±0.61	2.8±0.02
Linalool	2.14±0.31	1±0.15	11.42±1.85	13.97±2.09	1.05±0.08	-	-	-	-	15.58±2.64	-	-	2.05±0.3	0.99±0.05	-	-
2,4-Dimethylcyclohexanol	-	-	0.71±0.05	-	-	-	-	-	-	-	0.27±0.09	-	0.33±0.02	-	0.44±0.06	-
Terpinen-4-ol	1.64±0.39	0.55±0.04	2.51±0.24	1.59±0.34	0.47±0.03	-	0.19±0.05	-	-	1.26±0.21	0.73±0.04	0.35±0.05	-	0.77±0.01	-	-
1-Octanol,2-butyl-	-	-	-	0.54±0.1	0.4±0.03	0.21±0.01	-	-	-	-	0.99±0.18	0.2±0.01	-	-	-	-
**Esters**																
Butyl acetate	-	-	-	-	0.16±0.02	-	-	-	-	-	-	-	1.52±0.2	-	-	-
Vinyl caproate	-	12.24±1.65	-	-	0.25±0.08	-	-	10.91±2.05	11.77±1.6	-	-	-	0.48±0.02	-	0.19±0.04	0.77±0.13
Dimethyl glutarate	-	-	-	-	-	-	0.14±0.04	-	-	-	3.09±0.49	-	-	0.18±0.02	-	-
Ethyl phenylacetate	174.3±31.91	-	-	1.59±0.24	0.37±0.02	0.57±0.09	0.24±0.05	-	-	0.19±0.03	0.59±0.13	0.41±0.03	0.78±0.14	0.39±0.05	-	-
Ethyl caprate	-	-	-	-	-	-	-	-	-	0.18±0.02	0.2±0.05	-	-	-	-	-
Octyl salicylate	-	-	-	-	0.16±0.02	-	-	-	-	-	-	-	-	-	-	0.44±0.21
Methyl hexadecanoate	-	-	-	-	0.18±0.07	0.35±0.06	-	-	-	0.17±0.04	0.35±0.01	0.2±0.02	-	-	-	0.25±0.06
aldehyde																
Hexanal	3.74±1.28	-	2.48±0.67	4.16±0.46	4.38±0.46	0.99±0.25	1.13±0.21	-	-	1.99±0.25	1.61±0.07	1.53±0.12	-	1.12±0.26	1.75±0.13	3.24±0.37
Heptanal	0.63±0.08	6.43±0.71	0.99±0.09	2.27±0.11	0.5±0.04	0.35±0.07	1.66±0.09	1.97±0.3	6.16±1.13	2.33±0.51	0.93±0.23	1.27±0.04	1.04±0.11	0.71±0.01	2.25±0.02	3.63±0.53
Benzaldehyde	-	9.63±1.04	6.23±1.83	2.12±0.14	1.76±0.1	1.02±0.1	1.23±0.42	3.21±0.59	5.04±0.52	1.51±0.29	2.12±0.64	2.2±0.31	2.05±0.27	1.57±0.42	1.6±0.36	1.03±0.08
Octanal	10.23±1.62	12.76±1.32	4.86±0.89	13.66±1.39	2.97±0.35	2.34±0.11	5.68±1.02	3.77±0.87	14.96±2.32	9.38±2.01	23.75±3.03	7.07±0.31	-	-	7.54±0.4	11.49±2.45
2-Octenal, (E)-	-	7±0.42	-	2.19±0.15	0.25±0.01	-	1.13±0.36	2.22±0.42	4.79±0.83	0.82±0.26	1.89±0.29	0.76±0.2	-	0.86±0.27	-	-
Nonanal	5.57±1.18	21.52±2.99	7.46±1.15	12.02±0.86	6.84±1.15	4.62±0.46	10.42±1.79	9.35±1.61	23.07±3.47	14.03±2.72	10.78±2.33	11±0.84	9.6±1	7.34±1.56	13.56±0.64	19.21±0.23
Trans-2-nonenal	1.13±0.16	3.71±0.47	0.63±0.1	1.18±0.11	0.26±0.08	0.2±0.05	0.26±0.04	1.15±0.18	2.3±0.32	0.42±0.04	0.32±0.13	0.24±0.01	-	0.21±0.05	0.55±0.19	0.44±0
Decanal	0.98±0.21	3.24±0.28	-	173.16±1.85	0.13±0.01	-	0.22±0.03	0.42±0.06	1.45±0.17	0.79±0.21	0.84±0.21	0.21±0.04	-	-	0.43±0.02	0.46±0.02
4-Isopropylbenzaldehyde	-	0.55±0.07	2.27±0.26	24.97±3.02	6.53±0.72	0.65±0.12	0.17±0.08	0.55±0.06	6.16±1.24	0.8±0.07	1.96±0.4	2.24±0.07	0.96±0.19	-	0.5±0.09	0.33±0.02
trans-2-Decenal	-	3.02±0.31	0.74±0.03	1.31±0.19	0.3±0.04	0.32±0.09	0.33±0.01	0.62±0.07	1.25±0.23	0.51±0.08	0.57±0.11	0.44±0.05	0.57±0.09	0.31±0.07	0.48±0.15	0.45±0.07
2-Undecenal	1.62±0.34	2.31±0.16	0.56±0.04	0.81±0.06	-	0.35±0.06	0.24±0.03	-	-	0.31±0	0.29±0.06	0.33±0.01	0.44±0.07	0.27±0.06	0.33±0.09	0.4±0.05
Tetradecanal	1.39±0.38	1.67±0.45	0.57±0.2	2.88±0.55	2.27±0.1	-	1.97±0.42	-	-	1.39±0.37	3.07±0.57	2.68±0.43	2±0.5	3.44±0.28	1.2±0.24	0.35±0.07
Ketones																
2-Heptanone	2.73±0.59	1.43±0.35	-	1.86±0.31	0.9±0.11	-	-	-	-	-	1.41±0.28	-	-	-	-	0.37±0.06
Methylheptenone	42.99±9.08	-	-	-	-	-	-	-	-	0.61±0.1	-	-	0.8±0.07	-	-	-
2-Nonanone	4.4±0.41	3.14±0.27	0.55±0.06	0.94±0.15	0.2±0.02	-	0.25±0.08	0.39±0.05	0.96±0.17	0.7±0.15	0.64±0.16	0.19±0.02	-	-	0.42±0.09	0.67±0.01
Olefins																
β-Pinene	-	-	-	-	-	-	0.36±0.03	-	-	-	-	2.63±0.13	1.43±0.14	2.43±0.31	-	-
Limonene	10.84±2.12	2.2±0.11	25.68±3.54	6.37±1.04	-	1.07±0.01	0.6±0.02	-	17.98±3.19	1.33±0.25	3.16±0.86	0.64±0.03	21.7±1.53	4.49±0.56	0.24±0.06	0.27±0
g-Terpinene	70.31±13.45	-	2.19±0.32	-	0.19±0.01	0.4±0.11	0.12±0.03	-	1.28±0.35	0.82±0.22	6.01±1.57	3.61±0.31	1.29±0.13	0.29±0.05	0.23±0.01	-
Longifolene	-	-	0.61±0.04	0.6±0.07	-	-	-	-	-	-	-	0.67±0.06	5.44±0.88	-	-	-
l-Caryophyllene	1.11±0.26	0.53±0.06	3.05±0.32	6.5±0.9	-	0.21±0.02	-	-	-	0.61±0.09	1.7±0.39	-	-	1.78±0.26	-	-
2-Heptene	-	-	2.6±0.38	4.21±0.42	-	-	-	-	-	0.86±0.19	2.23±0.42	-	1.25±0.26	0.41±0.07	-	-
Alkanes																
Octane	-	0.52±0.06	-	-	-	-	0.2±0.01	-	0.42±0.06	0.36±0.06	-	-	-	0.38±0.03	0.86±0.09	0.79±0.14
Undecane	-	0.52±0.07	2.72±0.48	2.35±0.31	-	1.44±0.18	0.48±0	-	-	-	1.02±0.27	0.83±0.05	1.16±0.11	0.52±0.06	-	-
Tetradecane	-	-	-	-	0.22±0.01	0.28±0.01	-	-	-	-	0.26±0.06	0.21±0.02	0.27±0.04	33.87±4.42	-	0.25±0.08
1-Chlorooctadecane	-	0.5±0.05	-	0.63±0.09	-	0.17±0.02	0.14±0.04	-	-	0.26±0.05	0.26±0.09	0.18±0.02	0.68±0.11	-	-	-
Nonadecane	-	1.72±0.11	-	0.83±0.19	-	0.41±0.07	-	0.59±0.1	1.2±0.1	0.55±0.06	0.93±0.18	0.67±0.08	0.66±0.14	0.71±0.07	0.41±0.09	0.62±0.09
Acids																
Hexanoic acid	-	-	-	-	-	-	-	-	-	-	0.93±0.03	0.56±0.02	0.81±0.07	-	-	-
Dodecanoic acid, 3-hydroxy-	-	3.03±0.22	2±0.2	3.76±0.21	-	0.77±0.05	0.94±0.2	1.37±0.26	2.3±0.37	1.62±0.34	2.34±0.57	0.94±0.15	-	0.92±0.07	1.54±0.34	2.63±0.66

Pterin-6-carboxylic acid	3.06±0.64	-	-	-	0.22±0.02	-	-	-	-	-	0.47±0.1	-	-	-	-	-
Decanoic acid	-	-	-	-	0.11±0	-	-	-	-	-	0.28±0.06	-	-	-	-	-
Undecanoic acid	-	-	-	-	-	-	-	0.24±0.05	0.53±0.08	0.22±0.04	0.22±0.04	-	0.34±0.06	0.22±0.03	0.26±0.08	0.47±0.12
Othere																
2-Methylpyrazine	-	-	-	-	-	-	-	-	-	-	0.42±0.03	-	-	-	0.52±0.08	0.31±0.06
4-Methylpyrimidine	-	0.68±0.07	0.63±0.08	0.92±0.07	-	0.57±0.04	-	0.6±0.02	0.38±0	0.57±0.02	0.47±0.06	0.7±0.11	0.42±0.1	-	0.31±0.06	0.34±0.12
p-Xylene	-	-	-	-	-	-	0.15±0.04	-	0.41±0.1	0.21±0.05	0.24±0.06	-	-	0.62±0	-	-
2,6-Dimethylpyrazine	-	1.46±0.17	-	2.21±0.4	-	0.62±0.03	1.28±0.23	-	-	1.21±0.27	2.76±0.6	1.55±0.19	-	-	1.89±0.31	-
1-isopropyl-2-methylbenzene	-	-	-	2.14±0.41	-	-	-	-	4.13±0.86	-	5.17±0.94	1.82±0.16	0.64±0.04	-	-	-
2-Acetyl-3-methylpyrazine	-	-	-	-	-	-	0.14±0.05	0.27±0.05	-	-	0.23±0.02	0.15±0.03	-	-	-	-
Anethole	-	6.91±1.61	-	-	1.07±0.11	2±0.03	2.15±0.46	0.97±0.09	1.8±0.36	6.09±1.6	-	1.19±0.25	-	-	1.61±0.29	-

”-” means not detecte.
